# Origin of Stabilization of Ligand-Centered Mixed Valence Ruthenium Azopyridine Complexes: DFT Insights for Neuromorphic Applications

**DOI:** 10.1021/acs.jpclett.5c00812

**Published:** 2025-06-10

**Authors:** A. Avilés, S. Perez Beltran, M. Ghotbi, A. J. Ferguson, J. L. Blackburn, M. Y. Darensbourg, P. B. Balbuena

**Affiliations:** †Department of Chemical Engineering; ‡Department of Chemistry, and §Department of Materials Science and Engineering, 14736 Texas A&M University, College Station, Texas 77843, United States; ∥ Chemistry and Nanoscience Center, 53405 National Renewable Energy Laboratory, Golden, Colorado 80401, United States

## Abstract

Redox-driven conductance changes are critical processes in molecular- and coordination-complex-based memristive thin films and devices that are envisioned for neuromorphic technologies, but fundamental mechanisms of conductance switching are not fully understood. Here, we explore charge disproportionation (CD) processes in [Ru^II^L_2_]­(PF_6_)_2_ molecular systems that intrinsically involve interfragment charge transfer (IFCT). Using a combination of *ab initio* molecular dynamics simulation (AIMD), time-dependent density functional theory (TD-DFT), and density functional theory (DFT) calculations, we investigate the electron transfer mechanisms and the roles of temperature and cell volumetric expansion in facilitating the counterion movements and electronic transitions required for low-cost IFCT and charge redistribution. A detailed analysis of the density of states and TD-DFT calculations highlights that unpaired electrons play a crucial role in low-energy transitions, with the azo (N=N) groups of the ligand serving as the primary sites for electronic transport between molecular fragments, further stabilizing the asymmetric state. Localization of added electrons on azo ligands occurs with negligible change at the Ru centers, supported by atomic volume expansions up to +4.74 bohr^3^, and goes along with a progressive reduction of the HOMO–LUMO gap across redox states, suggesting enhanced conductivity. The TD-DFT analysis reveals a dominant IFCT excitation at 2082.76 nm in the doubly reduced (22) state, while a stabilization energy of 1.20 eV of the asymmetric (13) state relative to the symmetric (22) state is predicted by constrained DFT. Periodic DFT and AIMD simulations emulating a molecular film show that the stabilization of the asymmetric state, relative to a symmetric one, translates in net charge separation values (order of ∼0.33 e) that are strongly linked to increased counterion mobility (average counterion displacements exceeding 0.7 Å per atom during CD events) and the involvement of azo groups in electron redistribution. These findings, which align with previously reported experimental and computational data, provide key insights into the IFCT mechanisms and electronic transport facilitated by azo groups, with important implications for redox-driven memristive and neuromorphic technologies.

## Graphical abstract

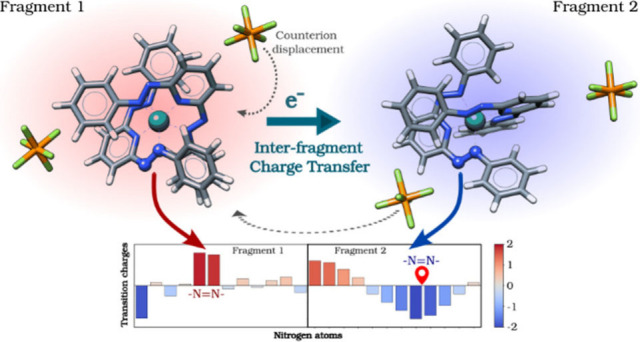

Several natural and artificial chemical and physical processes rely on the carefully choreographed movement of the electron density between specific sites to facilitate the desired functionality. The fact that these movements from one distinct landing site to another (oxidation/reduction) do not necessarily require in-deep thermodynamic valleys for information transmission is fundamental to biological neurons and energy-efficient artificial neuromorphic computing systems. Namely, the consequences of shifts in electron density, influenced by dynamic and fluctuating factors, are central to the neuromorphic function. The experimental and theoretical elucidation of the fundamental properties of individual molecules and molecular assemblies that are mimetics of neurons are at the heart of understanding and design of molecular neuromorphic analogues.^1,2^

Molecular materials such as organic semiconductors have recently generated considerable enthusiasm for computing applications due to their tunable electronic properties^3^ and easy solution processability.^4^ While some reports have highlighted organic memristors showing abrupt switching behavior,^5^ they have proven to be technologically irrelevant due to issues like poor reproducibility, limited stability (typically <1 h), and low durability (<10^3^ cycles), compounded by a lack of understanding of their underlying mechanisms. However, recent accounts suggest that many of these historical limitations may be overcome,^6^ allowing for more reliable use of molecular materials in advanced computing technologies.

Chemists can finely tune functionalities through molecular synthesis to offer a wide range of potential molecules for electronic applications in computing.^7–9^ This versatility has kept research in the molecular electronics field alive, despite technical obstacles and increasingly complex challenges.^10^ The widespread adoption of reliable organic light-emitting diodes in lighting and displays has also demonstrated that molecular films can indeed withstand large electric fields and current densities.^11,12^ Experimental techniques for *in situ* electrical and spectroscopic measurements show that molecular films can endure both processing and electrical operation.^13–16^ Alongside significant advancements of novel experimental techniques, theoretical developments and molecular simulations have improved considerably, allowing for a deeper understanding of molecular films and their interfaces.^17^ This progress has instilled confidence in materials scientists that they can successfully overcome the most challenging limitations of molecular computing, leading to a reassessment of the knowledge gained over the past three decades and driving a revolution in this field.^18^

Coordination complexes with organic ligands bound to a metal center have emerged as a prominent option.^19^ Their increased stability promises to address short lifetimes and thermal instabilities faced by organic semiconductors in electronic applications. And since both the ligand and metal ions are redox-active, their cooperation could enhance efficiency and selectivity in electron transfer processes. Thus, the resurgence in the chemistry of transition metal complexes with redox-active organic (typically di- and triazapyridinyl) molecules (ligands) and the intense exploration in this field have led to a new generation of organic molecular circuit elements,^13^ promising for molecule-based computing.^20,21^

Recent work by Goswami, Goswami et al.^10,13,17,19,22,23^ has featured several unique electrochemistry, spectroscopy, and device results that appear to reveal molecular properties advantageous to neuromorphic function and computing. Films of metal complexes containing azo-aromatic groups act as electron sponges, absorbing (reduction) or releasing (oxidation) electrons in response to the applied voltage. Such molecular redox processes have been reported to be robust, capable of withstanding over 10^12^ cycles without degradation.^22^ Charge disproportionation (CD) in these systems was proposed as leading to the formation of multiple stable, nonvolatile electronic states, suggested as crucial for memristors development. While these studies thoroughly assess several molecular analogues of the iconic Ru­(bpy)_3_^2+^ system, through solid state investigations, the detailed mechanism(s) by which information is transferred within molecular films remains elusive. Considerable challenges still present for integration of molecular materials into memristor devices^10^ demand a better understanding of the mechanisms that may operate at both the *molecular* and *ensemble* levels.

Taking a clue from the well-known approach for establishing mechanisms of enzyme catalysis by computational studies, we have extracted fragments of the full structure of [Ru^II^L_2_]^2+^ (L = a triaza tridentate ligand) as it is found in its PF_6_-salt (Figure 1).

**Figure 1:**
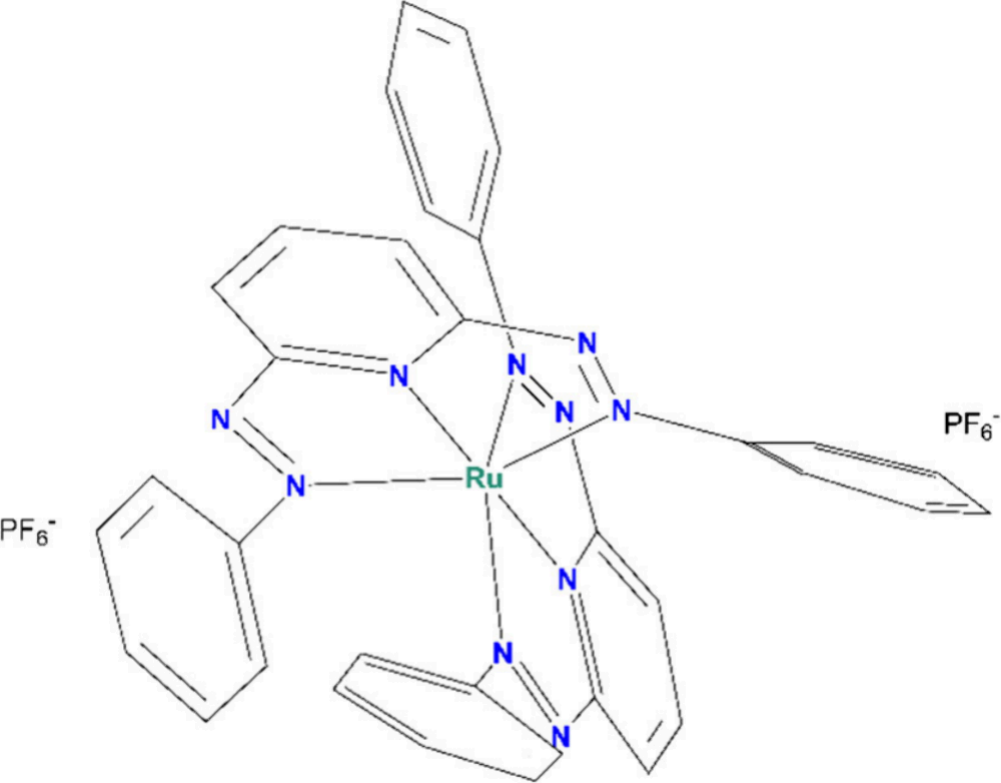
Molecular structure of [Ru^II^L_2_]­(PF_6_)_2_.

We use a combination of Ab Initio Molecular Dynamics (AIMD) simulations, Time-Dependent Density Functional Theory (TD-DFT), and Density Functional Theory (DFT) calculations to explore charge transfer between metal–ligand complexes, finding CD processes intrinsically involving interfragment charge transfer (IFCT). We define IFCT as a process involving charge transfer between molecules. We refer to each redox configuration of the complex as a specific “state”: state (0) denotes the neutral form, states (1) and (2) are the one- and two-electron reduced species of the molecule in Figure 1, respectively, and (11), (22), or (13) labels indicate dimers with distributed charges. Charge transfer processes are facilitated by cell volumetric expansion, enabling counterion movements and electronic transitions required for IFCT and charge redistribution. Our DFT analyses show that the azo groups of the ligands play a central role in electron transfer through this molecular complex. TD-DFT calculations provide critical insights into the specific interactions underlying IFCT. A systematic analysis reveals key electronic transitions at low energies, where unpaired electrons are the main contributors. The results show that the IFCT process allows the transition from a symmetric (22) electronic state to an asymmetric (13) state driven by α-electron transport between molecular fragments. Periodic DFT and AIMD simulations confirm the role of counterion dynamics on the stabilization of the (13) state relative to that of the (22) state.

## Methods

DFT is used to optimize the molecular geometry of the ruthenium complexes. Exchange–correlation effects were treated using the Perdew–Burke–Ernzerhof (PBE) functional.^24^ Gas-phase electronic structure calculations were carried out using Gaussian 16 software package.^25^ A mixed basis set was utilized, with LANL2DZ (Los Alamos National Laboratory 2 double ζ) for Ru and all-electron 6-31+G** for non-transition-metal atoms, which involves the use of an Effective Core Potential (ECP) for Ru.^26^ The objective of these calculations was to obtain the single-point energy after a global optimization, as well as to gain a better understanding of the nature of electronic transitions through TD-DFT level calculations,^27^ including Grimme’s D3BJ dispersion correction^28^ to obtain a more realistic description of both molecular packing and the energetic landscape relevant to the redox processes discussed in this work. The QTAIM charge analysis^29^ of the Ru complexes was conducted using the Multiwfn software.^30,31^ Molecular structure analysis, along with the visualization of iso-surfaces and maps derived from electron density, was performed using the UCSF Chimera software.^32^ The AV1245 index is a measure of aromaticity derived from the integrated electron density within a ring system. Higher values indicate greater π-electron delocalization, while lower values reflect reduced aromaticity. To facilitate interpretation, we note that the reference AV1245 index value for benzene is approximately 11.7 (in units × 1000).

To further explore the dynamic behavior of the Ru complexes, AIMD simulations were performed using the Vienna Ab initio Simulation Package (VASP).^33^ The system was modeled based on the X-ray crystal structure of [Ru^II^L_2_]­(PF_6_)_2_, with four molecules per unit cell. The initial structure was optimized using conjugate gradient minimization with a 4 × 4 × 4 γ-centered k-point grid and a plane-wave energy cutoff of 400 eV. Exchange–correlation effects were treated using the PBE functional, consistent with the DFT calculations performed in the gas phase.

The AIMD simulations were designed to explore how temperature and cell volumetric expansion affect the electronic and structural behaviors associated with the charge disproportionation process. AIMD simulations were carried out in the *NVT* ensemble over a total duration of 4000 fs, with a time step of 1.0 fs at constant temperatures of 240, 506, and 750 K, combined with simulation cell volumes with 0% and 30% expansions. The simulations included an equilibration period of 1000 fs to ensure system stability before collecting the data for analysis. Structure optimization of a cell containing four molecules M = [Ru^II^L_2_]­(PF_6_)_2_ in the (00) state was carried out using the conjugate gradient algorithm,^34^ allowing for the full relaxation of atomic positions. Additionally, stress tensor calculations were employed to optimize both the shape and volume of the cell.^35^ This approach ensures that both the internal structure of the molecules and the cell parameters are adjusted to minimize the total system energy, reaching the relaxed configuration of the atoms in their instantaneous ground state.

An optimized cell of the 4-molecule system was used as the starting point for AIMD simulations, with 8 electrons (average 2 e^–^ per molecule) then added to simulate the temporal evolution of the film with molecules in State (22). The initial simulation was performed in an *NVT* ensemble at a temperature of 506.0 K and a 30% expanded cell volume to facilitate counterion motion over a period of 10000 fs. The aim was to explore the role of [PF_6_]^−^ counterion movements in response to electronic changes that might favor the IFCT process, leading to the charge-disproportionated state (13).

During these exploratory simulations, the temperature was held constant for each expansion level to isolate the specific impact of volume on the electronic and structural behavior, particularly focusing on charge disproportionation. A Nosé–Hoover thermostat^36^ was used to maintain constant temperature control throughout the simulations. The postprocessing of the molecular dynamics simulations was carried out using the Python interface of the OVITO software,^37^ enabling detailed analysis and visualization of atomic trajectories and related properties.

Constrained DFT (cDFT) calculations using the NWChem software package^38,39^ were used in our computational protocol, to characterize the relative stability of a charge-disproportionated state (13). The cDFT methodology enables the enforcement of e^–^ localization constraints on specific fragments of the dimeric system, facilitating a direct comparison between symmetric and asymmetric charge distributions.^38^ This strategy was crucial to quantifying the relative energy of the (22) versus (13) states and to rationalizing the mechanistic aspects of charge transfer observed in the AIMD and TD-DFT analyses.

Figure 2 depicts AIM atomic charge distributions, reflecting how electron density is distributed in several of the electronic states observed experimentally^6,13^ in solution and film during operando Raman spectroscopy at different redox/conductance levels of [Ru^II^L_2_]­(PF_6_)_2_. Monomer electronic structure and frontier molecular orbitals agree with previous reports.^13^ Figure 2 describes the changes in AIM atomic charges within [Ru^II^L_2_]­(PF_6_)_2_. The electron density at the Ru metal center (Figure 2a) does not significantly increase in State 1 (1 e^–^ added). Negligible change in the Ru Bader charge suggests that the added electron is not localized on the metal. In contrast, a notable increase in electron charge is observed on the N atoms of the N=N ligand double bonds. The AIM charge of each N atom directly bonded to Ru increases from −0.47 to −0.49 e, while N atoms bonded to other N atoms (not directly to the metal) also increase in electron charge from −0.31 to −0.39 e. These changes indicate that most of the added electron density is distributed over these N = bonds, related to the ligand’s ability to participate in redox processes. It is important to note that no significant changes are seen in the AIM charges of *axial* N atoms (those bound to Ru in the heterocyclic ring), reinforcing the idea of the redox process centered on the azo (−N=N−) groups. The addition of a second electron, forming State 2, follows a similar pattern, with a slight increase in the Ru AIM charge and a more pronounced increase in electron charge on the N atoms of the N=N double bonds. The AIM electronic charge of N atoms bound to Ru changes from −0.49 to −0.52 e, and that of the noncoordinating N atoms in the azo bond increases from −0.39 to −0.45 e. This first analysis suggests that the N=N bond region is the main receptor site of the electron density during both reduction processes.

**Figure 2:**
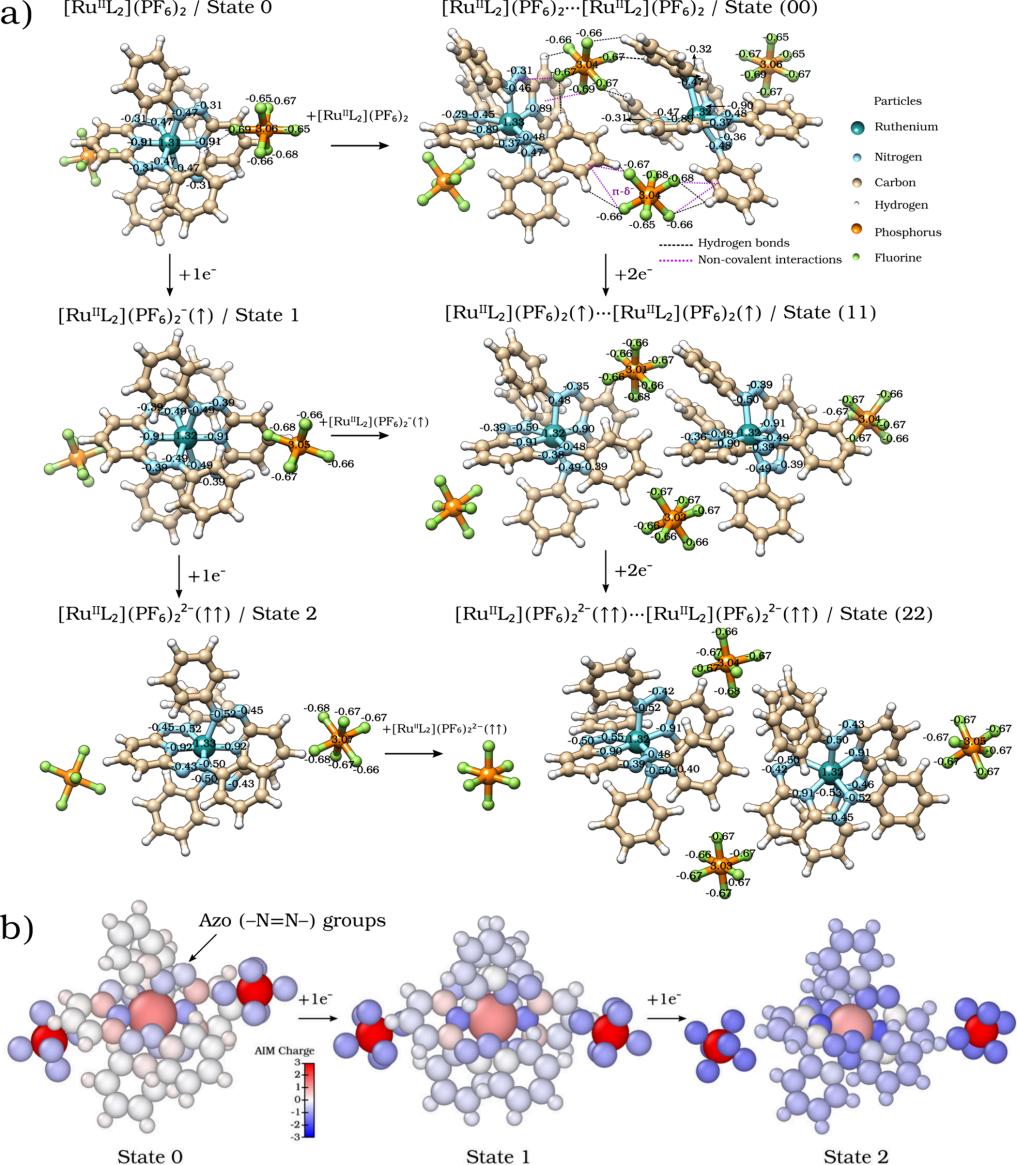
a) Optimized gas-phase structures at the PBE/LANL2DZ Level and AIM Atomic Charge Distribution for the [Ru^II^L_2_]­(PF_6_)_2_/State 0 complex during successive reduction processes and dimer formation. b) Color-coded representation of AIM atomic charge distribution for States 0, 1, and 2 of the [Ru^II^L_2_]­(PF_6_)_2_ complex, illustrating the evolution of electron density as additional electrons are introduced. The azo (−N=N−) groups in the ligand exhibit a pronounced increase in electron density (blue regions) during the transition between the states.

A combined analysis of the Electron Localization Function (ELF) and Nucleus Independent Chemical Shifts, [NICS]_ZZ_ values for the five-membered ring containing the azo (−N=N−) unit across redox states is shown in Figure S1-a. The 2D ELF plots reveal a progressive increase in electron delocalization between atoms of the azo-containing rings as the complex transitions from State (0) to State (2) and [NICS]_ZZ_ values computed at the ring geometric center decrease upon successive electron additions, suggesting gains of aromatic character upon reduction. Changes in AIM volumes, despite modest charge variations, reflect increased π-delocalization upon reduction, as further supported by ELF and NICSZZ analyses (Figure S-1).

Figure 2b uses a color-coded representation of charge density changes to provide a visual understanding of how the electron density evolves as additional electrons are introduced. The azo (−N=N−) groups in the ligand exhibit a significant increase in electron density (blue regions) as the system transitions from neutral State 0 to reduced States 1 and 2.

Phosphorus (P) and fluorine (F) atoms exhibit only minor changes in charge, suggesting that the [PF_6_]^−^ counterion does not play a primary role in the redox process. However, the interaction between [PF_6_]^−^ and the aromatic rings of ligand L in State 0, occurring via H-bonding (F–H distance of 2.12 Å), suggests a subtle contribution to the overall charge distribution. This interaction is accompanied by a charge shift in F (from −0.68 to −0.69 e) and in H (from +0.07 to +0.13 e), hinting at a possible charge transfer. These H-bond interactions disturb the electron density in the aromatic rings, reducing their aromaticity, as shown by the decrease in the AV1245 index^40^ by 0.45 in the phenyl ring and 0.67 in the heterocyclic ring (calculated using QTAIM partitions, with AV1245 values multiplied by 1000). The AIM charges of the carbon (C) atoms exhibit minor variations upon reduction. A slight decrease in the AIM charge of P, and the reduction in the total AIM volume of [PF_6_]^−^ support the notion of partial charge transfer from the counterion to the cation. Additionally, the observation that most F atoms lose electron density when forming State 0 from isolated [PF_6_]^−^ and [Ru^II^L_2_]^+2^ reinforces this subtle role. While the counterion’s involvement in the redox process is limited, its response to the redox process contributes to fine-tuning the charge distribution.

The location of the redox-active region of the Ru complex in the N=N double bonds is further supported by the results in Table 1, where AIM volume changes are shown with the addition of two electrons. The largest volume change, +4.74 bohr^3^, occurs in the N atoms of the N=N bonds (not directly bonded to Ru), which matches the observed electron charge increases. In contrast, the volume changes in the C, H, P, and F atoms are much smaller. Also, the slight decrease in the AIM volume of Ru further suggests that the metal atom is not directly involved in the redox activity. This analysis allows us to establish that the role of the Ru center in this complex is predominantly structural, while the redox activity is concentrated in the ligands.

**Table 1:** Change in AIM Charges (e) and Volumes (bohr^3^) between Monomers (State 0 vs 2) and Dimers (State 00 vs 22):

Element	ΔAIM charge (State 0 vs 2)	ΔAIM volume (State 0 vs 2)	ΔAIM charge (State 00 vs 22)	ΔAIM volume (State 00 vs 22)
Ru	+0.021	–1.88	–0.002 to −0.01	–1.83 to −1.89
N	–0.0136 to −0.115	–0.81 to +4.74	–0.019 to −0.0613	+1.79 to +6.89
C	–0.0298 to +0.0038	–1.34 to +2.17	–0.0516 to +0.0037	+1.08 to +1.61
H	–0.0403 to −0.0471	+3.19 to +2.6	–0.0296 to +0.0079	+6.12 to +1.95
P	+0.01273	–0.18	–0.004 to −0.007	+0.6 to +0.61
F	+0.0085 to −0.01796	+3.4 to +3.3	+0.0164 to −0.0134	+6.2 to +3.7

While State 0 shows a symmetrical distribution of charges across the ligands, particularly noticeable in the N atoms coordinating the metal and those involved in the azo (−N=N−) groups (Figure 2a, top row), when two molecules in State 0 interact, this symmetry breaks (State 00) due to electron density redistribution. In State 00, the Bader charges of the axial N atoms change from −0.91 to −0.89 e, and the ∠N–Ru–N angle decreases from 178.6° to 167.0°. Despite this, the strength of the Ru–N­(axial) bond remains largely unchanged, as indicated by Wiberg bond order values of 0.88 in State 0 and 0.89 in State (00).

On the other hand, the equatorial N atoms involved in the N=N bonds exhibit varying AIM charges, ranging from −0.45 to −0.48 e. In State 00, some of the N atoms in the double bonds not directly bonded to the metal gain more electron charge (−0.37 e compared to −0.31 e in State 0), suggesting a tendency for electron density to accumulate in this region through attractive interactions between units. The Wiberg bond order for N=N in State 0 is 1.63, while in State (00), the N=N bond with the highest electron accumulation (charges of −0.48 and −0.37 e) has a bond order of 1.57. Similar to the dimer formation process, the Wiberg bond orders for the N=N bonds also decrease when two electrons are added to State 0 to form State 2, dropping from 1.63 to 1.42 and from 1.60 to 1.47. These reductions in Wiberg bond orders indicate a progressive weakening of the N=N bonds as the electron density increases in this region of the ligand.

Figure 2 shows the redistribution of atomic charges during the reduction of the dimer State (00). When State (00) receives two electrons (one per molecule), forming State (11), the Ru centers exhibit minimal change in charge, with only one Ru atom slightly affected. The added electron density localizes primarily on the N=N double bonds of the ligand, as indicated by an increase in the AIM charges of the nitrogen atoms from −0.37 to −0.47 e in State (00) to −0.49 to −0.50 e in State (11). Figure S-2 (Supporting Information) supports this, showing that in the triplet state (11), the α-spin density is concentrated on the N=N bonds, while the Ru centers remain largely uninvolved. This conclusion holds for the (0) → (1), (1) → (2), (0) → (00), (00) → (11), and (11) → (22) processes.

Two [Ru^II^L_2_]­(PF_6_)_2_ complexes interacting in the gas phase is shown as State 00 (Figure 2a), with two [PF_6_]^−^ counterions positioning themselves between the metal–ligand complexes, forming noncovalent interactions with both. These interactions include F–H hydrogen bonds with the aromatic systems as well as a π–δ^–^ interaction (π-system-induced dipole) between the F atoms and the π clouds of the aromatic rings of the L ligands in both complexes. Additionally, attractive noncovalent interactions are recognized between the F atoms and the C=C double bonds of the phenyl groups in the L ligands (Figure 2). Notably, the distance between the F and H atoms of the phenyl rings ranges from 2.04 to 2.62 Å, further reinforcing the presence of H-bonding. Similarly, π–δ^–^ interactions between the F atoms and the π-electron systems of the phenyl rings occur at distances of around 2.47 Å, contributing to the electronic stabilization of the system. These noncovalent interactions were recognized through a topological analysis of the electron density using the real space function Interaction Region Indicator (IRI),^41^ shown in Figure S-3. These interactions, while not altering the fundamental electronic structure of the [Ru^II^L_2_]­(PF_6_)_2_ complex, serve to fine-tune the electron distribution across the system. The slight electron density accumulation observed in some regions, particularly around the N=N bonds, can be attributed in part to these counterion interactions. This redistribution contributes to the overall stabilization of the dimeric form, allowing the system to accept additional electrons without substantial structural reorganization.

The [PF_6_]^−^ counterions exhibit a minimal redistribution of charge after the reduction processes, particularly in the F atoms. In State (00), the charges of the F atoms range from −0.65 to −0.67 e, while in State (11), these values undergo slight changes (−0.66 to −0.68 e). This suggests that the counterions maintain a predominantly electrostatic interaction with the metal–ligand complexes and do not absorb a significant amount of electron density during the reduction. Additionally, the average Ru–P distance (between Ru and the [PF_6_]^−^ counterions) increases from 5.66 Å in State 00 to 6.86 Å in State 11. Similarly, elongations are observed in all distances associated with noncovalent interactions between the F atoms and the atoms of the aromatic groups in the ligands.

As the reduction progresses, in State (22), the average Ru–P distance increases significantly to 8.11 Å, compared to 6.86 Å observed in State (11). This increase in distance is accompanied by a highly symmetrical arrangement, where the four P atoms of the [PF_6_]^−^ counterions are nearly coplanar, forming a dihedral angle ∠P–P–P–P of 178.50°. In the global energy minimum of State (22), the two [Ru^II^L_2_]^2+^ metal cations exhibit a similar spatial orientation with the L ligands aligned in a comparable fashion. The [PF_6_]^−^ counterions are also symmetrically positioned around the metal centers, reinforcing this structured configuration. The observed symmetry in the atomic arrangement likely contributes to the transient structural stability of State (22), as the noncovalent interactions and electrostatic forces between the metal–ligand complexes and counterions are uniformly distributed, promoting a relative stability in the system despite its metastable nature. This metastability is further discussed in the next section based on the analysis of the frontier molecular orbitals.

Based on the analysis of the Electron Delocalization Range Function (EDR)^42^ (see isosurfaces in Figure S-3), a progressive increase in electron delocalization is observed as the system transitions from a monomeric configuration (State 0) to a dimer (00) and then undergoes the reduction processes. In State 0, the EDR isosurface shows no interaction between the surfaces of the metal–ligand complexes and those of the counterions. However, in the dimeric state (00), these surfaces completely overlap, indicating total delocalization of the valence electrons throughout the system. A key aspect is how this delocalization evolves during the successive reduction processes, particularly when comparing State (00) to State (11) (Figures S-2 and S-3). A clear increase in electron delocalization is detected, as two additional electrons are added during the reduction. This phenomenon is especially relevant in the dimer as the increased electron delocalization following electron addition would be expected to facilitate better charge carrier mobility. The electronic interactions between the metal–ligand complexes and the counterions become more coherent and extended, potentially reducing the resistance to electron flow. This change in electron distribution could correlate with an increase in the material’s electrical conductivity, potentially explaining prior conclusions^13^ of the (00) conductance being low and the (22) conductance being considerably higher.

Figure 3 depicts the calculated frontier molecular orbitals (FMOs) and associated energy levels for States (0), (00), (11), and (22), obtained via unrestricted DFT. In all redox states, the FMOs are predominantly localized on ligand π* orbitals, consistent with experimental cyclic voltammetry^13^ and prior theoretical analysis. For State (0), the presence of four unoccupied π* orbitals (LUMO to LUMO+3) enables successive one-electron reductions, in accordance with Hund’s rule (Figure S-4). The HOMO–LUMO energy gap in the isolated [Ru^II^L_2_]^2+^ monomer (1.38 eV) is only marginally affected by the inclusion of [PF_6_]^−^ counterions (1.36 eV), indicating a negligible perturbation of the FMOs. Counterions primarily act as electrostatic stabilizers without altering the system’s electronic structure. Compared to the monomeric State (0), the dimerized State (00) displays a reduced HOMO–LUMO gap, decreasing from 1.36 to 1.19 eV. This narrowing arises from the emergence of noncovalent interactions, specifically H-bonding and π–δ contacts, between the two [Ru^II^L_2_]^2+^ metal–ligand complexes and the [PF_6_]^−^ counterions in State (00), which enhance interfragment electronic coupling and stabilize the dimeric assembly. The FMOs energies shift modestly, and no notable changes are observed in orbital topology or phase, indicating that dimerization does not qualitatively modify the MO character.

**Figure 3:**
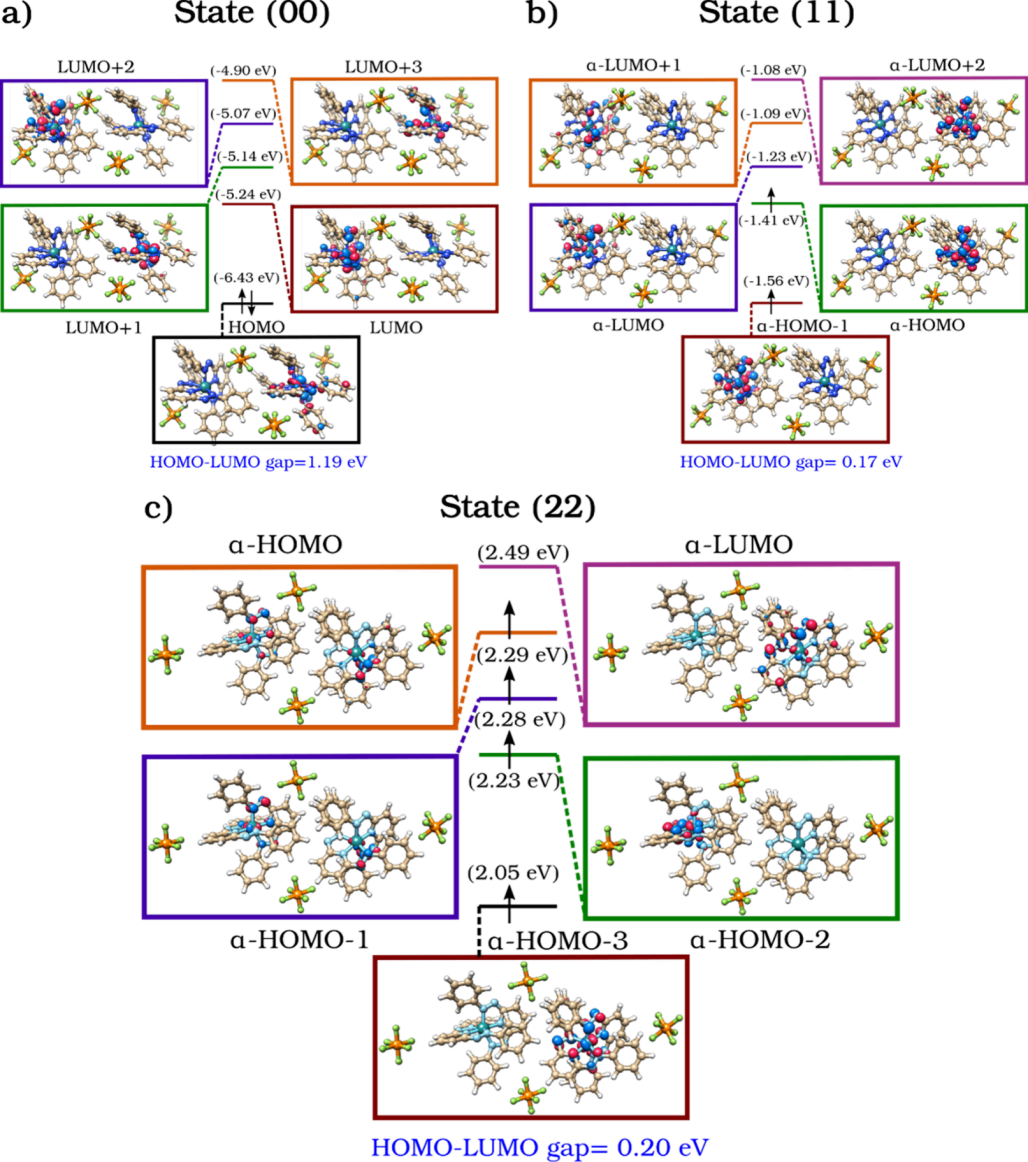
Comparison of HOMO–LUMO energy levels in a) State (00), b) State (11) and c) State (22), showing the effects of counterions and electron addition. HOMO = highest occupied molecular orbital; LUMO = lowest unoccupied molecular orbital.

Upon adding two electrons to State (00) to form State (11), the α-HOMO and α-HOMO–1 orbitals become partially occupied, corresponding in character to the former LUMO and LUMO+1 orbitals of State (00) (Figure 3b,c). These orbitals shift to higher energies (−1.56 and −1.41 eV, respectively), indicating that the added e^–^ populate less stabilized, ligand-centered states. Despite this energetic shift, the spatial character of the FMOs remains unchanged, and the spin density is primarily localized on the ligands. The resulting triplet state displays one unpaired e^–^ per metal–ligand fragment, with minimal delocalization toward the Ru centers.

The energy gap between α-HOMO and α-LUMO in State (11) is reduced to 0.17 eV (Figure 3c), compared to 1.19 eV in the neutral dimer (Figure 3b). This marked narrowing of the frontier orbital gap enhances the probability of low-barrier electronic transitions, thus promoting intramolecular conductivity and responsiveness to external stimuli in the reduced state.

This marked contraction in the frontier orbital gap suggests a greater propensity for e^–^ transport in the reduced (11) system. The smaller energy gap in state (11) should not only enhance the system’s intermolecular conductivity but also allow for a greater responsiveness to voltages and other external stimuli.

Comparison between States (11) and (22) reveals pronounced reorganization within the FMOs, indicative of substantial electronic restructuring in the doubly reduced state. Specifically, the α-HOMO and α-HOMO–1 orbitals of State (11) evolve into α-HOMO–2 and α-HOMO–3 in State (22), exhibiting marked energy shifts to 2.23 and 2.05 eV. Notably, the newly formed α-HOMO and α-HOMO–1 orbitals in State (22) are nearly isoenergetic (2.29 and 2.28 eV), delocalized across the two [Ru^II^L_2_]­(PF_6_)_2_ fragments. This delocalization suggests increased electronic coherence and interfragment communication in the (22) system, which could enhance charge mobility and improve conductivity in this state. Among all spin configurations examined, only the high-spin quintet (^5^State (22)) exhibits the key electronic features associated with the CD mechanism—namely, IFCT and delocalized FMOs across both metal–ligand complexes. These characteristics are absent in the lower-spin states (Figure S-5), indicating that the high-spin configuration is the only electronically relevant state for enabling e^–^ hopping. While the (22) state was not experimentally observed in the molecular film by Goswami et al.,^13^ it has been detected in solution, implying that solvation may stabilize its electronic structure.

State (22) exhibits a distinctive feature in its positive energy values for the FMOs, suggesting lower electronic stability compared with states (00) and (11). A distinctive feature of State (22) is the unusually high energy of its FMOs, pointing to lower electronic stability relative to States (00) and (11). This situation indicates that the second reduction process could lead to an intermediate (22) state with a shorter lifespan, making it more susceptible to electronic changes. State (22) is valence-symmetric, with each fragment containing two unpaired electrons in antibonding orbitals, and is distinguished by the presence of a singular azo vibrational mode. These elevated energy levels indicate that the additional electrons are in less stabilized positions within the system, clearly indicating a potential predisposition toward charge disproportionation. In this scenario, the symmetric (22) configuration may break symmetry under external stimuli via an asymmetric redistribution of electron density, leading to a mixed-valence state where one [Ru^II^L_2_] unit becomes more oxidized or reduced than its partner.

The intrinsic electronic instability of the high-energy FMOs in State (22) suggests that it already constitutes a broken-valence intermediate. In this configuration, asymmetric charge redistribution between the two metal–ligand complexes becomes energetically favorable, leading to localized mixed-valence character. This picture aligns with the proposed formation of State (13), where one Ru^II^L_22_ unit becomes singly reduced and the other triply reduced: [Ru^II^L_2_]­(PF_6_)_2_^–^(↑)···[Ru^II^L_2_]­(PF_6_)_2_^3–^(↑↑↑), as described by Goswami et al.^6,13^ Thus, State (22) represents an electronic configuration at the edge of the CD and symmetry breaking.

To investigate the potential instability of the (22) state, Figure 4 displays the most probable electronic transitions calculated for the (22) uniform redox state by using scalar time-dependent DFT. These TD-DFT calculations are critical for evaluating the potential of the (22) state to undergo charge disproportionation, potentially transitioning to a more stable state such as (13).

**Figure 4:**
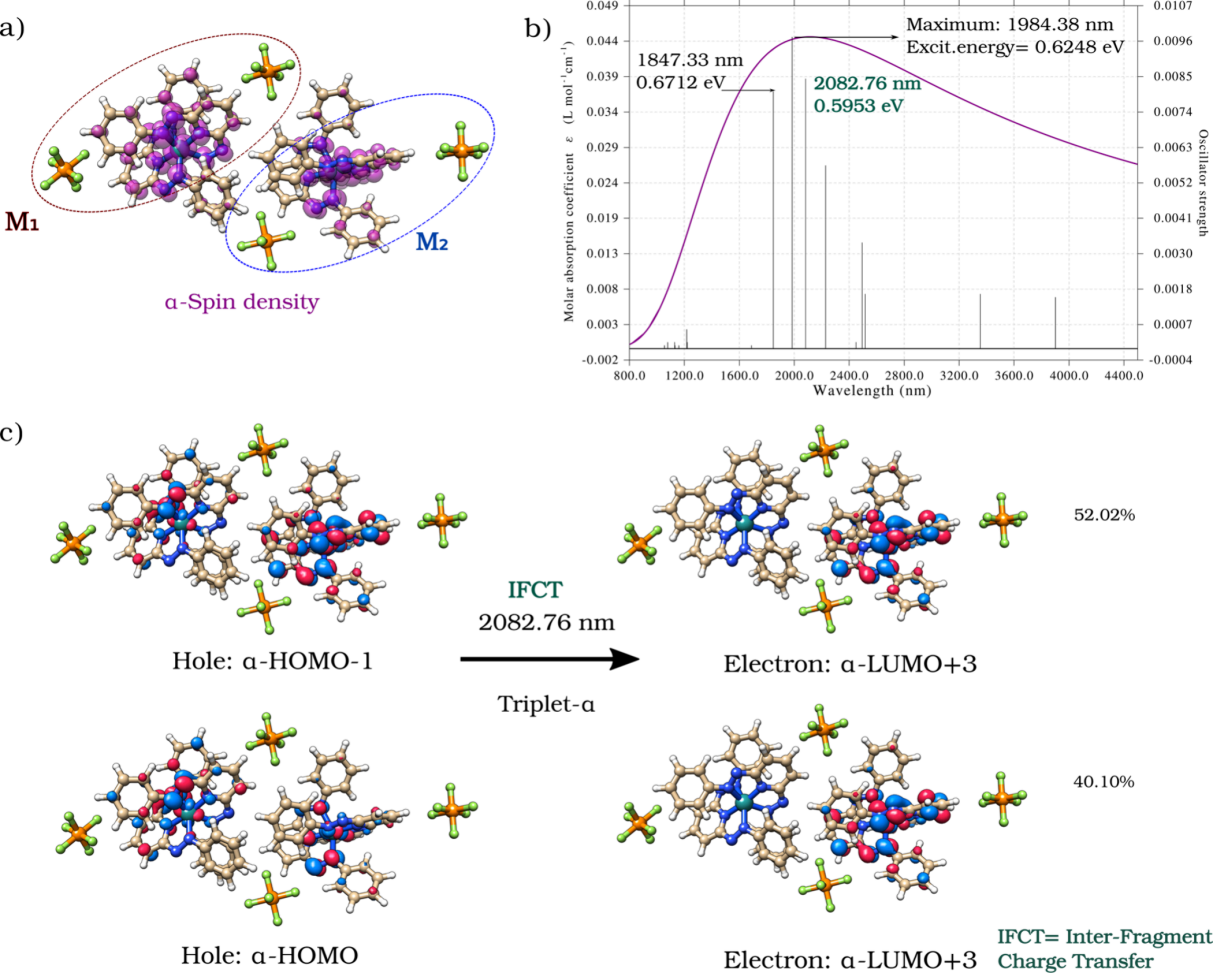
a) Structure and spin density of States (22) represented with an isosurface value of 0.002 au (e^4^/bohr^4^) (dark pink). b) Theoretical TD-DFT UV–vis spectrum for a pair of doubly reduced molecules, M_1_ and M_2_, associated with the symmetric and uniform redox state (22). c) Relative contributions of each transition to the total excited state, providing insight into the symmetry breaking that leads to the formation of the asymmetric (13) state through charge disproportionation in the film.

A high-spin (HS) configuration was considered in this analysis. The calculated maximum absorption peak appears in the near-infrared (NIR) region at 1984.38 nm/0.6248 eV and corresponds to a triplet α-transition from α-HOMO–2 to α-LUMO+3 [95.39%], exhibiting a mixed MLCT/LLCT character (MLCT = metal-to-ligand charge transfer; LLCT = ligand-to-ligand charge transfer), and is associated with an intrafragment excitation process (Figure 4).

The second prominent absorption band appears at 2082.76 nm/0.5953 eV and corresponds to a triplet-α IFCT transition with mixed MLCT/LLCT character. The relative contribution of each electronic transition to the total excited state is detailed in Figure 4c and can be described as follows: α-HOMO–1 (MO delocalized over both molecular fragments M_1_ and M_2_) → α-LUMO+3 (MO localized on M_2_) [52.02%], and α-HOMO (delocalized over M_1_ and M_2_) → α-LUMO+3 [40.10%].

According to this theoretical description, in the (22) state, the α electron from M_1_ can be promoted from the HOMO or HOMO–1 orbitals (which are delocalized over both molecular fragments) to LUMO+3, which is clearly localized on M_2_. This transition facilitates the process of charge transfer from component M_1_ to component M_2_, which can be interpreted as an electronic charge transport process from M_1_ to M_2_. These two electronic transitions (52.02% and 40.10%) allow M_1_ to lose an electron (by donating to LUMO+3 in M_2_) and M_2_ to gain that electron, establishing a mechanistic pathway for charge disproportionation. This pathway leads the system from a symmetric (22) state, where both components have two unpaired α electrons, to an asymmetric (13) state, in which M_1_ loses an electron and M_2_ gains one, resulting in 1 and 3 unpaired electrons, respectively. This electron transfer through the LUMO+3 localized on M_2_ creates an asymmetric charge distribution, where M_2_ accepts more charge and M_1_ becomes partially oxidized. This process stabilizes the system as it progresses toward the (13) state, minimizing the total system energy through electronic redistribution.

The absorption peak at 2082.76 nm suggests a coupled transition between molecular vibrations and electronic movements, where the electronic charge transfer is accompanied by adjustments in the vibrational modes of the molecular fragments and may also involve counterion movements. This is important because it suggests that the charge transfer process between M_1_ and M_2_ in the (22) state occurs smoothly, without crossing potential energy surfaces, maintaining the system in its quintuplet configuration. This possibility is explored in depth in the section discussing AIMD simulations for the unit cell of the (22) system. This vibro-electronic coupling is common in MLCT/LLCT transitions involving changes in charge distribution and molecular geometry,^43–45^ as seems to be the case in the (22) state.

Experimental results under field cooling (FC) conditions^13^ show that the system becomes trapped in a bistable configuration between the (00) and (11) states below 175.0 K, while the (13) state remains inaccessible due to the energy barrier associated with counterion displacements. This behavior aligns with TD-DFT calculations for the redox state (22), which predict that the transition to a more stable, asymmetric state, such as (13), requires overcoming an energy barrier for the IFCT to occur. The identified electronic transitions suggest that the (22) state can stabilize by absorbing energy (0.5953 eV) and initiating a charge disproportionation process. Furthermore, since TD-DFT calculations for the (11) state indicate that all identified transitions are intrafragment and do not involve IFCT (Figure S-6), this highlights a clear limitation in the ability of the (11) state to induce the necessary charge redistribution to directly reach the (13) state. The absence of IFCT suggests that the (11) state lacks the electronic characteristics needed to promote significant charge transfer between molecular fragments. This emphasizes the need for an intermediate state to facilitate this charge redistribution where the (22) state plays a crucial role.

In this context, the transition from the (11) state to the (22) state can be understood as a process involving the injection of additional electrons into the M fragments. In the FC experiments, cooling was performed with a + 8 V bias applied to the (00) state, and this applied bias marked the start of the voltage cycle. Mechanisms such as applying an electric field and/or electron transfer from the electrode—facilitated by an increased temperature above 175.0 K with the consequent rise in system conductivity—contribute to this electron injection. In the (11) state, the electrons are confined in bonding orbitals, but in the (22) state, the additional electrons occupy antibonding orbitals in each molecular fragment (and, in some cases, are delocalized across both fragments), destabilizing the system and increasing electronic mobility. As electrons are injected into the (11) state and the counterions adjust to compensate for changes in electronic charge, the stabilization of the (22) state is further promoted with (22) representing a local minimum on the potential energy surface (PES). The symmetry of the (22) state and its enhanced capacity to promote interfragment charge transfer make it a critical intermediate, enabling the charge disproportionation necessary to form the asymmetric (13) state. Additionally, we evaluated the relative stability of the asymmetric (13) state compared to the symmetric State (22) using cDFT calculations. These calculations reveal that State (13) is energetically more stable by 1.20 eV relative to State (22). This significant energy difference underscores the thermodynamic preference for charge disproportionation under the appropriate conditions, further supporting the hypothesis that the (22) state serves as an intermediate state.

To elucidate the underlying details of the charge transfer mechanism, we performed additional analyses in relation to the processes shown in Figure 4. The transition charge analysis presented in Figure 5 provides further insights into the electronic transport occurring during the triplet-α IFCT process that connects the (22) and (13) states. As the α electron is transferred from M_1_ to M_2_, Mulliken atomic charge analysis reveals a clear redistribution of electron density between the molecular fragments. Figure 5 focuses on how this electronic transport evolves during the adiabatic transition in the gas phase. The results are visualized as heat maps, showing the transition charges for M_1_ (left) and M_2_ (right). Positive charges, representing electron density loss, are colored red, while negative charges, corresponding to electron density accumulation, are shown in blue. Our findings indicate that electron transfer predominantly occurs from one of the N=N double bonds in M_1_ (N atoms with atomic indices 18 and 63, highlighted in red) to two N=N double bonds in M_2_. The first double bond in M_2_ involves N atoms with indices 104 and 116, while the second bond is formed by N atoms with indices 103 and 148. These N=N bonds are on two different ligands, suggesting that the electron that is transferred is uniformly distributed across both of the ligands in the acceptor complex. This redistribution of charge highlights the asymmetric nature of the charge transfer, driving the transition from the symmetric (22) state to the asymmetric (13) state.

**Figure 5:**
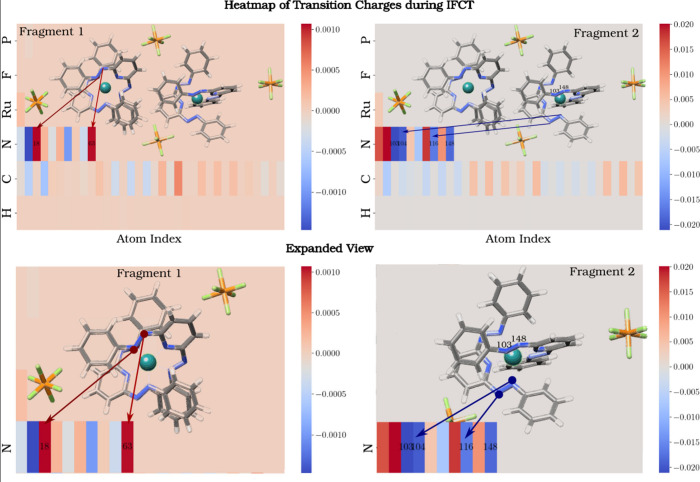
Transition atomic charges during interfragment charge transfer (IFCT) from State (22) to excited State (13), showing the redistribution of electron density during the initial step of charge disproportion. The heat maps display positive atomic charges in red and negative charges in blue. Each small rectangle represents an individual atom, while each row corresponds to all atoms of a specific element present in the system. At the bottom, an expanded view highlights the charge transfer regions within each molecular fragment.

The heat maps also show that the C atoms do not exhibit significant changes in their transition charges, as reflected in low-intensity areas. This indicates that the C atoms do not actively participate in the charge redistribution during IFCT between states (22) and (13). This suggests that electron transport is concentrated on the N atoms, potentially indicating that the C framework primarily serves a structural role rather than being directly involved in the electronic activity. This redistribution of charge highlights the asymmetric nature of the charge transfer, driving the transition from the symmetric (22) state to the asymmetric (13) state.

It is particularly interesting to note that while two of the azo (−N=N−) groups in the ligand of the molecular fragment M_2_ gain electron density (with negative transition charges), the other two azo (−N=N−) groups show a marked loss of electron density (with positive transition charges). This behavior indicates a nonhomogeneous redistribution of electron density within the ligand, where certain regions act as centers for electron acceptance, while others become polarized to donate charge.

To address the potential for device implementation of molecular film assemblies, we evaluated the organization and dynamics of larger systems. Thin films of transition metal complexes for neuromorphic devices are typically fabricated by dissolution of the complex into an appropriate solvent and subsequent spin-coating of this solution onto a substrate. Film thickness can be controlled by the solution concentration, spin-coating conditions, and substrate surface treatments, and thicknesses ranging from a ca. 10 nm to several hundreds of nm have been quoted for prior memristor studies.^46^ The thin films tend to have densities that are appreciably lower than that of the corresponding single crystal and do not exhibit the long-range crystallinity that is easily observed for single crystals via X-ray diffraction (XRD).^47,48^ Instead, there are likely local regions of varying size with crystal structures similar to that of crystals but of insufficient size to be probed by XRD measurements. In the model film composed of M molecules, where [PF_6_]^−^ counterions are present, a 30% expansion of the simulation cell has been chosen to represent the lower density of the molecular arrangement in the film. The choice of 506.0 K in the AIMD simulations aims to accelerate dynamic processes, enabling more efficient exploration of the transition pathways between electronic states, particularly the charge disproportionation leading to the (13) state. Additionally, this higher temperature helps overcome energy barriers that could slow down the IFCT processes, allowing for a deeper exploration of the potential energy landscapes. Figure 6a illustrates the atomic distribution within the cell at 3527 fs, showing the relative configurations of M1 and M3. To monitor the subtle positional shifts of the [PF_6_]^−^ counterions during this simulation, the average displacement of the P nuclei in the [PF_6_]^−^ ions was tracked over time using the equation Δ*P*(*t*) = ∑_*i*=1_^*n*_*p*_^Δ*P*_*i*_(*t*), where *ΔP*_*i*_(*t*) are the displacements of the center of mass of the P atomic positions for each of the counterions. Variations in *ΔP*(*t*) were examined to evaluate the relationship between the average anionic displacement and the previously described electronic transition pathways. This is particularly relevant when considering that State (22) is not detected in a quasi-solid film but is observable in solution,^13^ where the mobilities of the counterions are expected to differ significantly. Additionally, we can infer *a priori* that the mobility of the counterions in the film is highly correlated, more easily leading to a uniform state. This is depicted in Figure 6a,b.

**Figure 6:**
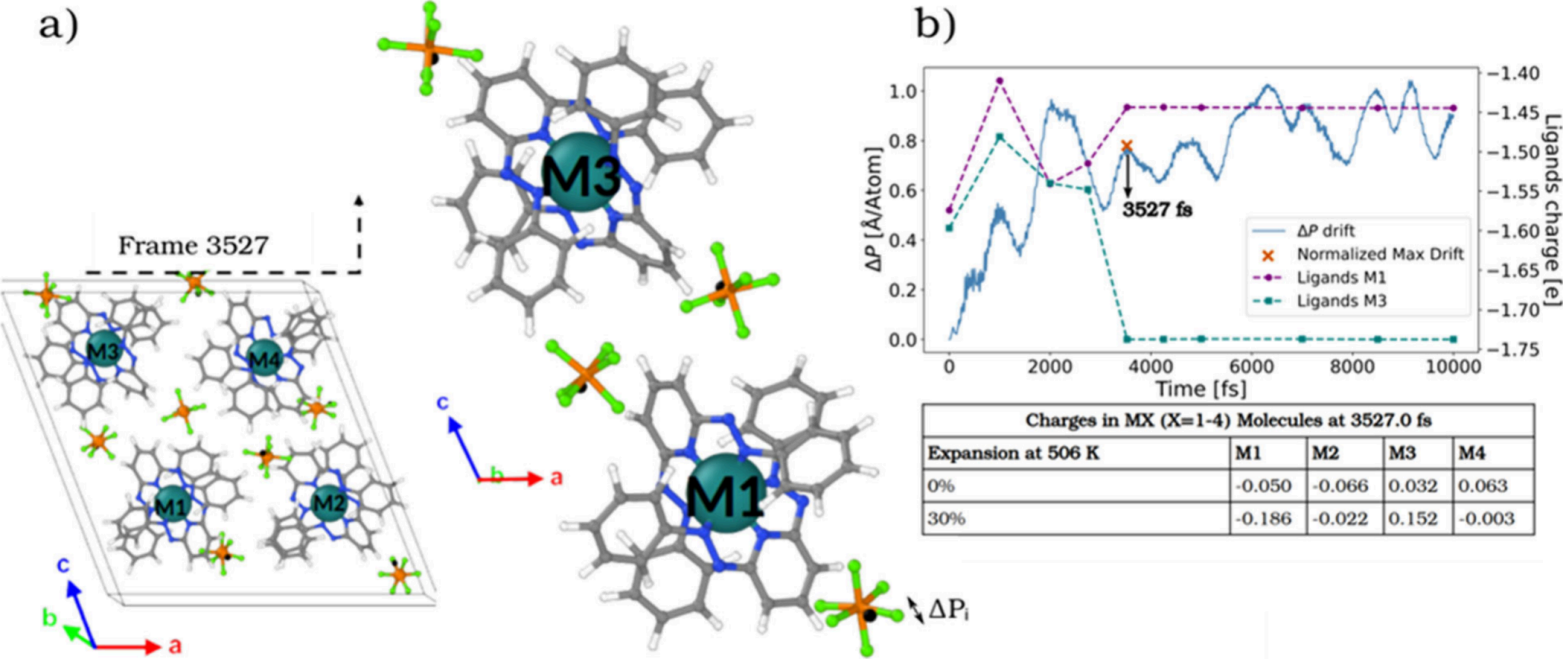
a) Cell structure and atomic distribution of four molecules M = [Ru^II^L_2_]­(PF_6_)_2_ in State (22) at the key event time of 3527.0 fs. b) Average counterion displacement ΔP­(t) during AIMD: Evidence of IFCT between M1 and M3. Superimposed curves show the total AIM charges of ligands coordinated to M1 and M3, revealing a clear IFCT. The black arrow at 3527 fs highlights a point where counterion displacement correlates with charge disproportionation.

Figure 6b highlights a relative maximum displacement observed at 3527.0 fs, coinciding with a CD event that could be linked to the formation of the asymmetric (13) state. At this moment, the charges of M1 and M3 are 0.144 and −0.1868, respectively, suggesting a clear charge transfer from M1 to M3. We therefore observe that M1 is partially oxidized as it loses electronic charge, while M3 is reduced as it gains electrons. This event occurs at a local maximum in counterion displacement, indicating a strong correlation between counterion movements and the redistribution of the electronic charge. Notably, after 4000 fs, the rate of counterion displacement begins to level off or fluctuate around a stable value, possibly implying that a threshold has been reached where further counterion movement is limited by factors such as stabilization of the electronic structure. Furthermore, the time-resolved AIM charges of the ligands (Figure 6b), provide compelling evidence of a CD event at 3527 fs. The M1 and M3 ligand charge profiles clearly diverge over time: the pair coordinated to M3 becomes increasingly reduced, while those bound to M1 are oxidized. This redistribution involves both ligands per coordination sphere. The fact that this IFCT occurs exactly at the peak of counterion displacement further supports the proposed coupling between nuclear motion and electronic reorganization.

The calculated spin density of states (DOS) of the neutral and 8 e^–^ added systems for the simulations in Figure 6 are shown in Figure S-7. The DOS reveals that α-spin unpaired electrons dominate the transition region near the Fermi level, driving the (22) → (13) transformation. No significant β-spin density is observed near the Fermi level at any point during the simulation. This is an important distinction: if a broken symmetry singlet (22) configuration were present, we would expect β-spin states near the Fermi energy, enabling β-mediated transitions—yet this is not observed. Therefore, the DOS data suggest that the (22) state in our system behaves effectively as the ^5^State (22) from the onset, prior to its conversion into the asymmetric CD state.

In addition to the 4-molecule simulation, another AIMD simulation was performed for a cell containing 8 molecules per unit cell and 16 additional electrons under the same conditions of 506.0 K and a 30% volume expansion. These results (Figure S-8), further validate the CD event observed in the smaller system.

To gain insight into the longer-time dynamics of the system and the role of [PF_6_]^−^ counterions in facilitating IFCT, a Bader charge analysis was performed on an AIMD trajectory at 750 K for an optimized cell in the State (22) containing 4 molecules and 8 additional e^–^s. The analysis revealed that when counterions remain equidistant from Ru_2_ and Ru_4_ (e.g., at 1457 fs; Frame A in Figure S-9), a balanced charge distribution is maintained between molecules M_2_ and M_4_ (Figure S-9, bottom). In contrast, an asymmetric arrangement, such as that observed at 4688 fs (Frame B) results in CD, with M_2_ acquiring a charge of −0.119 and M_4_ acquiring a charge of 0.168. This behavior is further illustrated by the charge maps (Figure S-9, top right), where reddish and bluish regions highlight the loss or gain of electronic density, signaling valence symmetry breaking. The correlation between counterion proximity and charge imbalance is consistent across multiple frames (e.g., 1249, 3773, and 4107), and mirrors the geometry of the cDFT-optimized gas-phase structure of State (13) (Figure S-10), where the Ru center of metal complex moiety carrying one up e^–^ lies closer to the counterions than the one carrying three. These findings emphasize that counterion dynamics (especially the selective approach toward a specific [Ru^II^L_2_]^2+^ unit) can modulate the electronic landscape, promote the IFCT process, and stabilize the asymmetric, charge-disproportionated State (13).

This study provides essential insights into CD dynamics, understood as a process that necessarily involves IFCT in the [Ru^II^L_2_]­(PF_6_)_2_ molecular system. The asymmetric nature of the charge redistribution demonstrated in this work could have significant implications for the overall electronic behavior of the system. Rather than merely serving as a passive electron reservoir, the ligand actively participates in modulating electron transfer between the metal–ligand complexes. The dual behavior of different N=N double bonds—some acting as electron donors and others as acceptors—implies that the ligand could function as a switchable conduit for charge transfer and as a gateway for electron flow, dynamically facilitating or regulating the charge transport process between M_1_ and M_2_, as well as the electronic coupling between both metal centers. This selective redistribution suggests the ligand’s capacity to create dynamic, tunable pathways for electron flow, potentially having a profound impact on the material’s overall conductivity and its response to external stimuli. The ability of certain N=N double bonds to switch between electron-accepting and -donating roles introduces flexibility that may stabilize the system during charge disproportionation, promoting the transition from the symmetric (22) state to the asymmetric (13) state. This active modulation by the ligand highlights a more intricate interplay of electron transfer mechanisms that could be harnessed for applications in molecular electronics and memristive devices, where controlled charge transport is the key.

On the other hand, the [PF_6_]^−^ counterions act as dynamic mediators, whose relative positions and displacements enhance electronic mobility between the molecular fragments, promoting IFCT. This synergistic interaction between counterions and ligands underlines the importance of precise molecular and device design to optimize charge transport mechanisms in advanced redox-based neuromorphic systems.

## Supplementary Material



## Notes

ELF maps and NICSzz values for redox states (0), (1), and (2) (Figure S-1); visualizations of electron density, α-spin density, ALIE, Laplacian, EDR, Fermi hole, and IRI for various molecular and dimeric electronic states (Figures S-2 and S-3); comparison of HOMO–LUMO levels between [Ru^II^L_2_]^2+^ and State 0; TD-DFT UV–vis spectra for the singlet and triplet forms of State (22) and TD-DFT UV–vis spectrum of State (11), including molecular orbital contributions to the excitations (Figures S-4 to S-6); spin DOS of the 4-molecule cell in neutral and 8 e^–^ added states (Figure S-7); AIMD simulations of State (22) configurations showing counterion motion and its role in triggering IFCT and charge redistribution (Figures S-8 and S-9); cDFT-optimized gas-phase geometry of asymmetric State (13), highlighting spin density and Ru–counterion distances (Figure S-10); quantitative analysis of AIM charge differences as a function of counterion proximity, evidencing the link between spatial arrangement and CD (Table S-1) (PDF)
