# Supplementary material for: Origin of Stabilization of Ligand-Centered Mixed Valence Ruthenium Azopyridine Complexes: DFT Insights for Neuromorphic Applications
Source: J Phys Chem Lett. 2025 Jun 10;16(24):6125–37. doi: 10.1021/acs.jpclett.5c00812 (PMC12193119; doi:10.1021/acs.jpclett.5c00812)
Supplement: Supplementary file 1 [file jz5c00812_si_001.pdf]

Supplementary Information

Origin of Stabilization of Ligand-Centered Mixed Valence Ruthenium Azopyridine Complexes: DFT Insights for Neuromorphic Applications

A. Avilés, *[1] S. Perez Beltran,[1] M. Ghotbi,[2] A. J. Ferguson,[4] J. L. Blackburn,[4] M. Y. Darensbourg[2], P. B. Balbuena[1,2,3,*]

[1]Department of Chemical Engineering; [2]Department of Chemistry, [3]Department of Materials Science and Engineering, Texas A&M University, College Station, Texas 77843

[4] Chemistry and Nanoscience Center, National Renewable Energy Laboratory, Golden, Colorado 80401

*e-mails: aaviles@tamu.edu; balbuena@tamu.edu

Computational Methodology for Electron Density-Based Analyses

All complementary analyses based on the electron density were performed using Multiwfn 3.8(dev). [1,2] These include the computation and visualization of electron density, its Laplacian, the Fermi hole, and the Interaction Region Indicator (IRI) to identify bonding and noncovalent interaction domains. The Electron Delocalization Range Function (EDR) was used to explore the extent of multicenter delocalization within redox states, while the Average Local Ionization Energy (ALIE) was mapped onto the electron density to highlight reactive sites and electronic stabilization patterns.

The AV1245 index, a multicenter aromaticity descriptor and previously introduced in the main text [64], was computed using QTAIM partitions to quantify aromaticity variations. For comparison, the AV1245 value for benzene is ~11.7 (×10³). All visualizations are shown in Figures S-1 and S-2, with corresponding isovalues indicated in the captions and were selected based on standard literature practices for clarity and comparability.

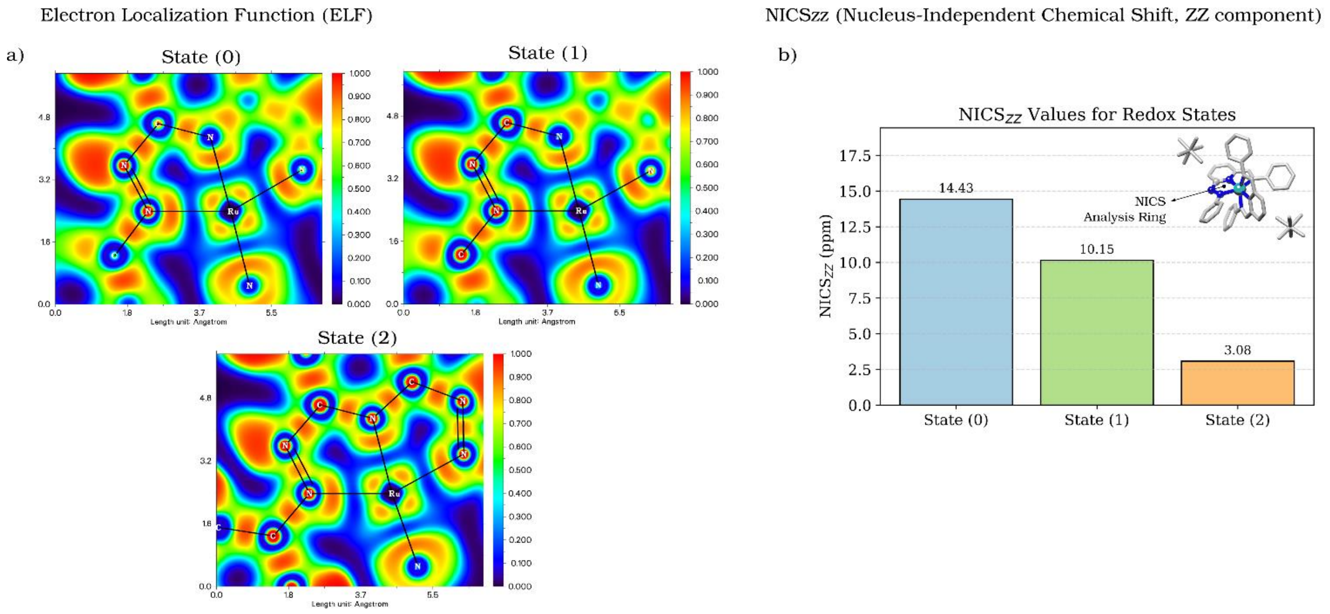

Figure S-1. a) Electron Localization Function (ELF) maps projected onto the molecular plane containing the Ru atom and the azo groups for redox States (0), (1), and (2). Red-colored internuclear regions (ELF ≈ 1.0) denote high electron localization and enhanced π-delocalization across the azo-containing rings upon reduction. b) Bar graph of NICSzz values (in ppm) calculated at the center of the five-membered azo-containing rings in each redox state. The progressive decrease in NICSzz values reflects an increase in local aromaticity, further supporting a ligand-centered reduction mechanism.

ELF and NICSzz Analyses

The Electron Localization Function (ELF) [3] and Nucleus-Independent Chemical Shift (NICSzz) [4] were computed using Multiwfn (v3.8).[3,4] ELF color-filled maps were projected onto the molecular plane containing the Ru center and the azo moieties using 200 grids in the two dimensions to visualize changes in π-delocalization across different redox states. NICSzz values were evaluated at the center of the five-membered azo-containing rings as a magnetic aromaticity descriptor, with the Z component taken along the direction normal to the ring plane. The magnetic shielding tensors required for the NICSzz calculations were obtained via Gaussian 16 using the NMR=GIAO keyword.

As shown in Figure S1-a, the 2D ELF plots reveal a progressive increase in electron delocalization between atoms of the azo-containing rings as the complex transitions from State (0) to State (2). Specifically, we observe that the internuclear regions within the five-membered ring become more intensely red (ELF ≈ 1.0), suggesting enhanced localization of π-electron density. In State (2), all atoms of the two symmetry-related rings lie in the same plane, and the continuity of red ELF regions between them supports the formation of an extended conjugated π-system.
To complement this topological analysis, $NICS_{ZZ}$ values were computed at the geometric center of the ring. Interestingly, the values decrease upon successive electron additions: State (0): 14.43 ppm, State (1): 10.15 ppm, State (2): 3.08 ppm. This trend indicates a gradual loss of paratropicity (associated with antiaromaticity), consistent with a system gaining aromatic character upon reduction. Together, the ELF maps and $NICS_{ZZ}$ data confirm that the azo-containing rings evolve from moderately antiaromatic to π-delocalized and nearly aromatic as the system is reduced. No significant changes were observed in the local ELF environment around the Ru center across the three states. This observation confirms that the redox events are predominantly ligand-centered, with minimal perturbation to the electron density surrounding the metal site.

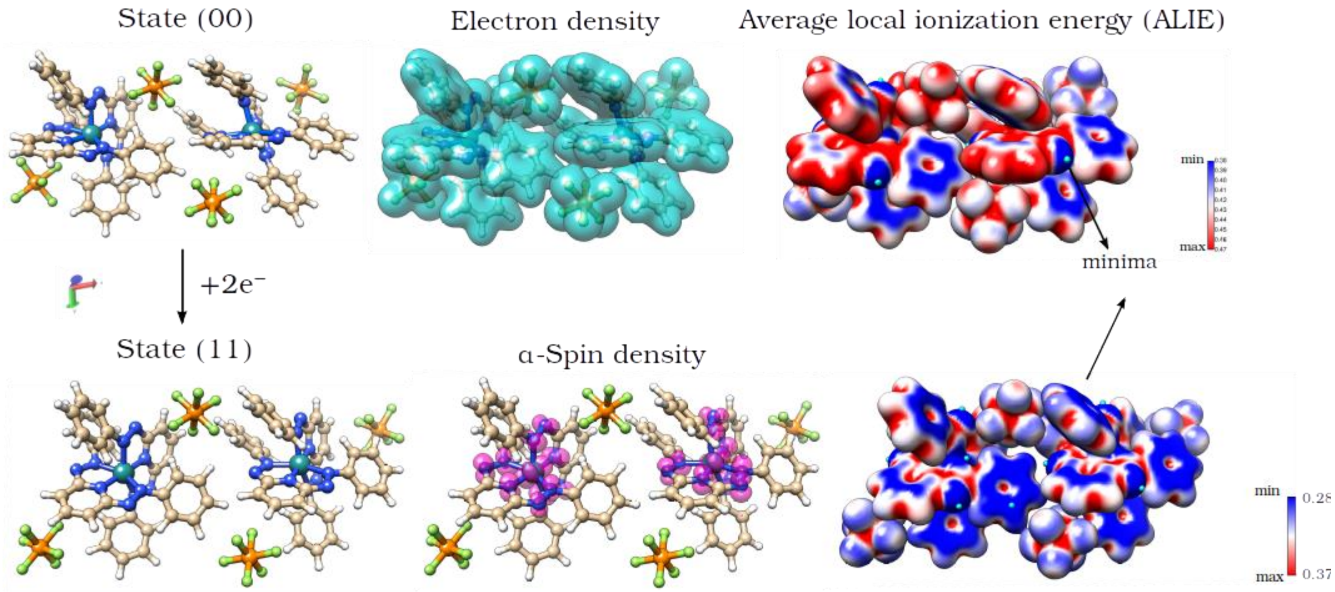

Figure S-2: Comparative Visualization of Electron Density (isovalue 0.01), α-Spin Density (isovalue 0.002), and Average Local Ionization Energy (ALIE, isovalue 0.012) for State (00) and State (11)

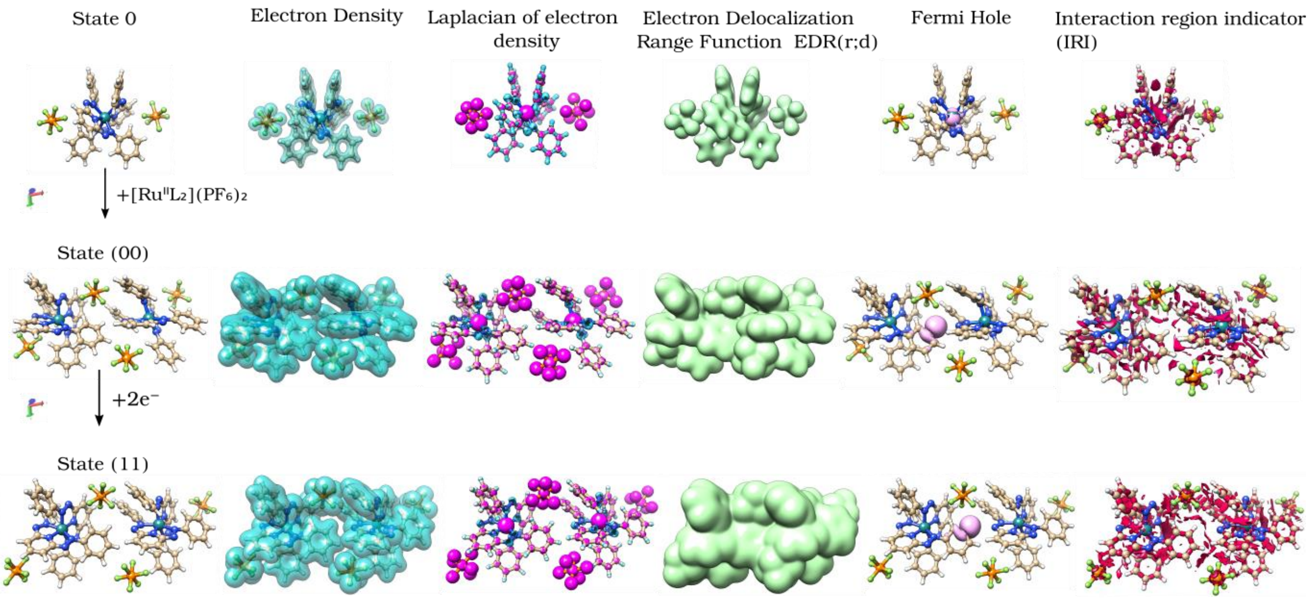

Figure S-3: Isosurfaces of Electron Density, Laplacian of Electron Density, Electron Delocalization Range Function (EDR), Fermi Hole, and Interaction Region Indicator (IRI) for States 0, (00), and (11).

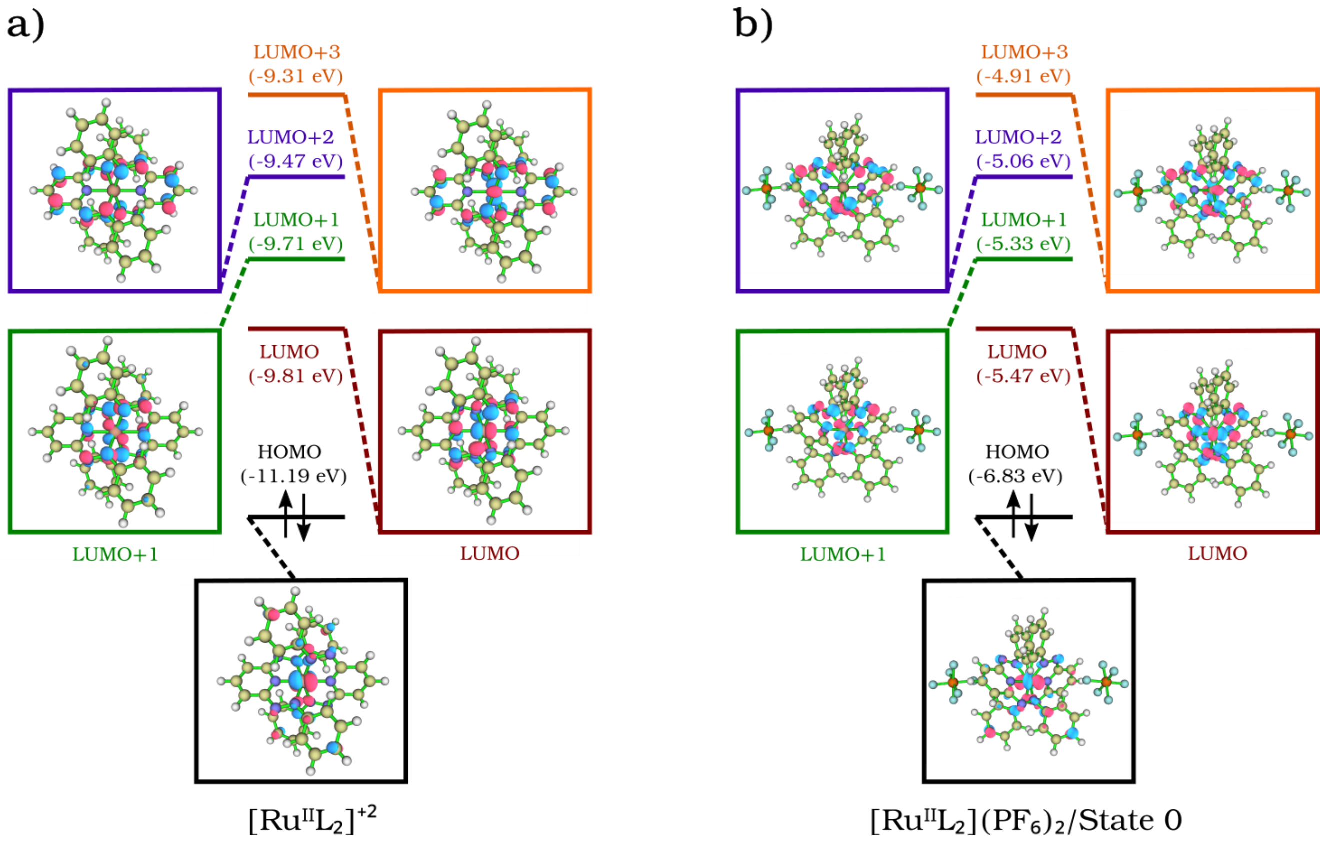

Figure S-4: Comparison of molecular orbital energy levels in the HOMO-LUMO region between the isolated $[Ru^{II}L_2]^{2+}$ complex and the $[Ru^{II}L_2](PF_6)_2$/State 0 complex with counterions.

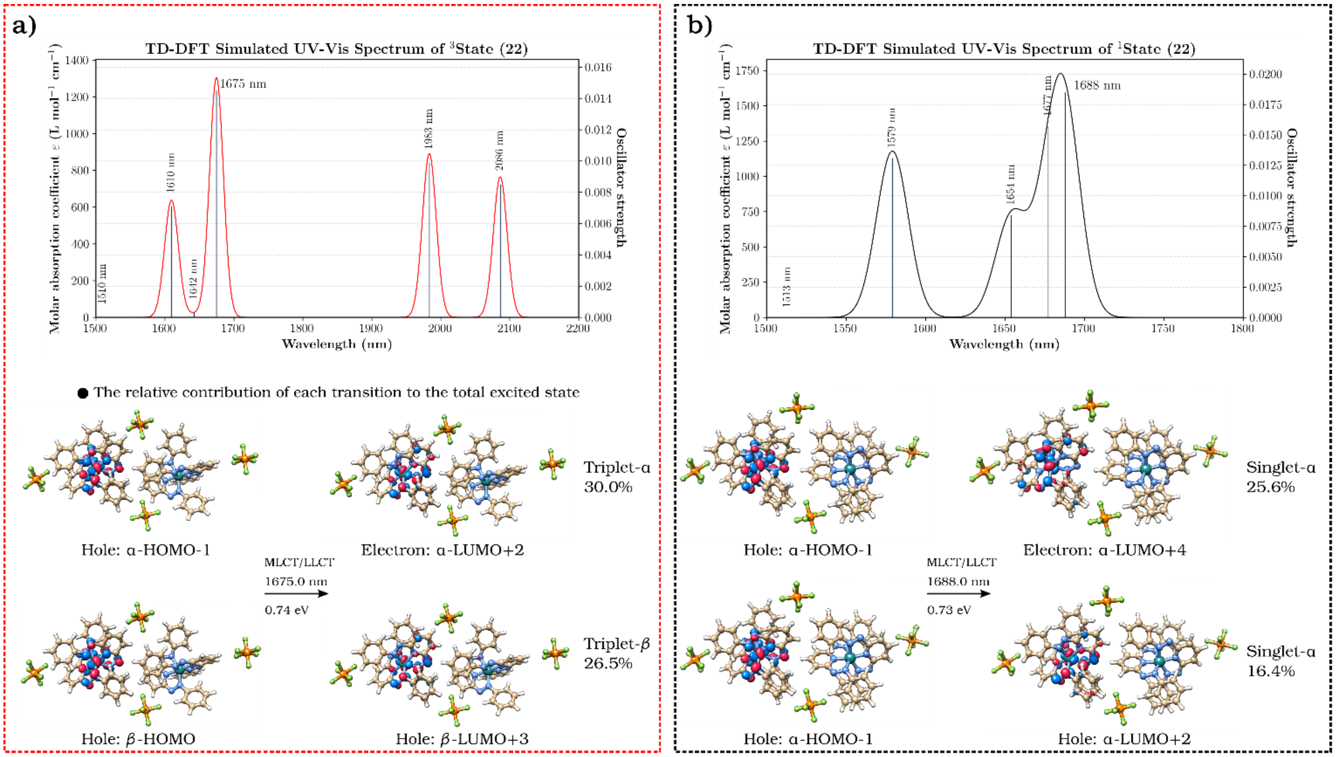

MLCT/LLCT= Mixed character Metal-to-ligand charge transfer/Ligand-to-ligand charge transfer

Figure S-5. TD-DFT simulated UV-Vis absorption spectra for the low-spin singlet [^{1}State (22), right] and

intermediate-spin triplet [^{3}State (22), left] states of the [$Ru^{II}L_2$](PF_6)$_2$ complex. The most intense peak in each spectrum is analyzed, and the relative contributions of the dominant electronic transitions to the total excited state are indicated. All excitations were fully characterized and involve intra-fragment electron transfer, with mixed metal-to-ligand (MLCT) and ligand-to-ligand (LLCT) character. No inter-fragment charge transfer was observed in either case.

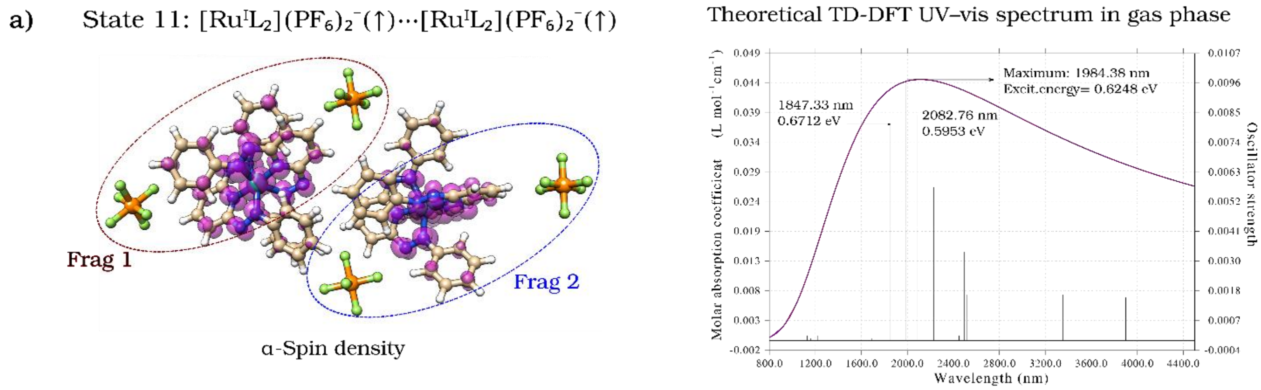

b) The relative contribution of each transition to the total excited state

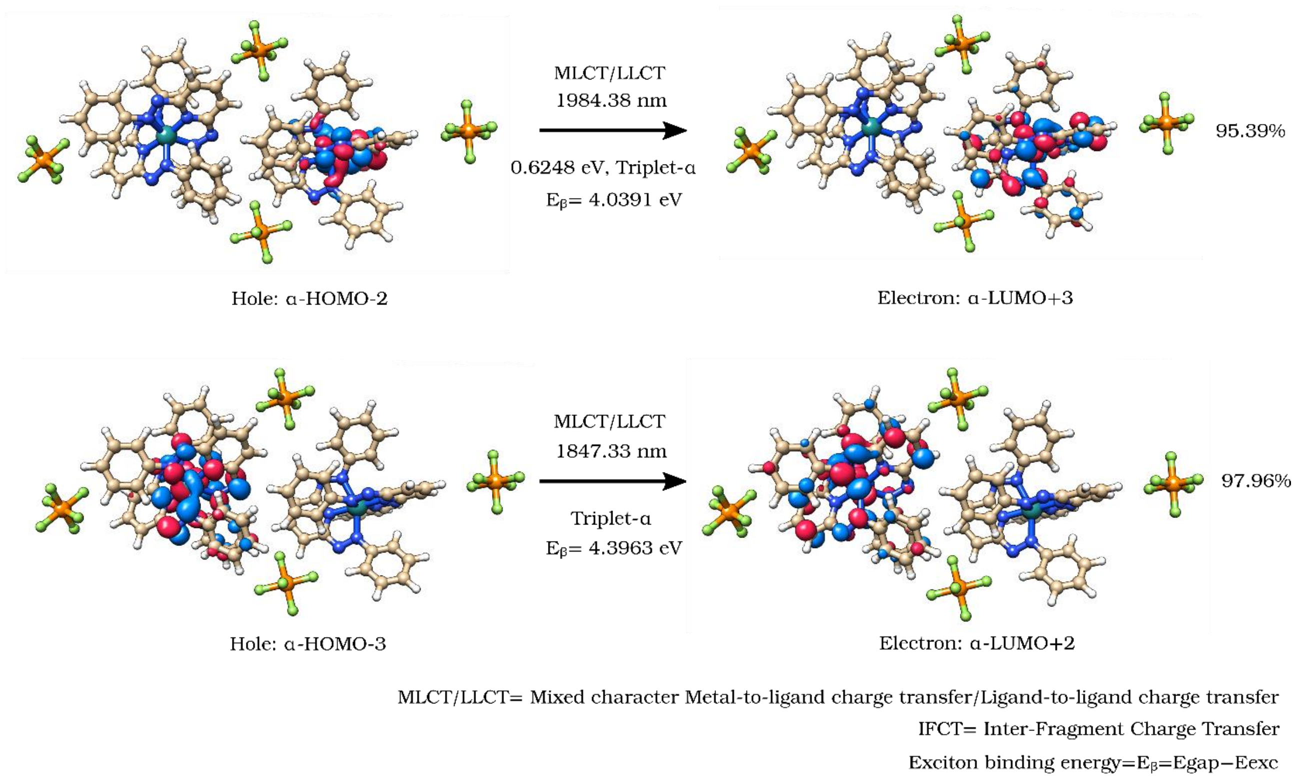

Figure S-6. a) Spin density of State (11) at 0.002 au ($e^4/bohr^4$) isosurface value (dark pink). b) Theoretical TD-DFT UV–vis spectrum of State (11) in the gas phase. c) MO representations of the electronic transitions from the TD-DFT absorption spectrum, with the relative contribution of each transition to the excited state.

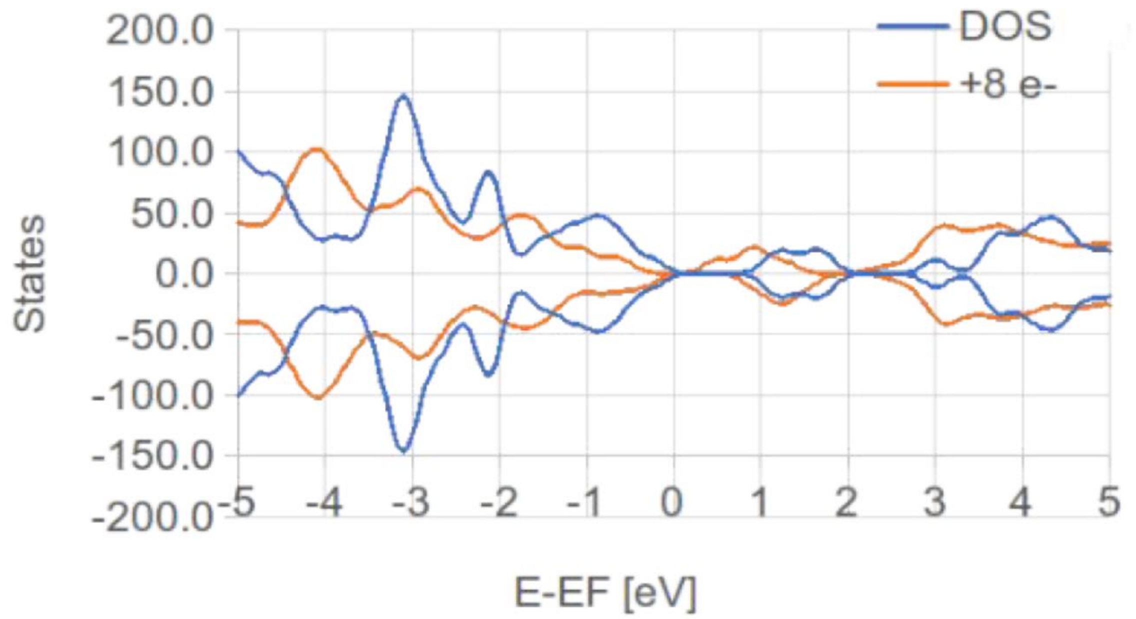

Figure S-7. Spin-resolved density of states (DOS) for the 4-molecule system with 8 additional e⁻, immediately after the CD event. The blue curve represents the α-spin DOS, showing a strong localization of unpaired electrons near the Fermi level, while the orange curve corresponds to the total DOS, reflecting a broader distribution of states. Plotting both together highlights that α-spin electrons dominate low-energy transitions and drive the (22) → (13) transformation.

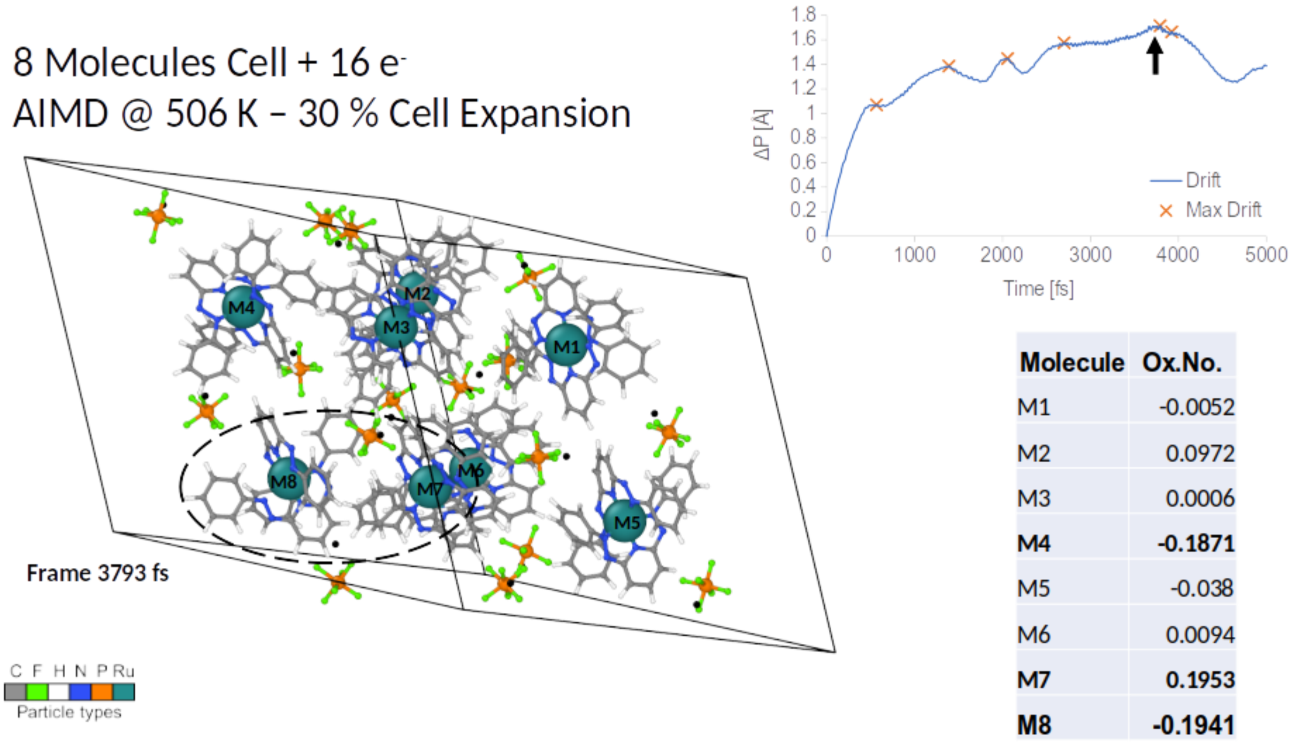

Molecule	Ox.No.
M1	-0.0052
M2	0.0972
M3	0.0006
**M4**	**-0.1871**
M5	-0.038
M6	0.0094
**M7**	**0.1953**
**M8**	**-0.1941**

Figure S-8. a) Atomic distribution and cell structure of eight M = [Ru^{II}L$_2$](PF$_6$)$_2$ molecules in the (22) state at the key event time of 3793.0 fs. b) Average counterion displacement ΔP(t) during AIMD simulation: Clear evidence of interfragment charge transfer (IFCT) between M$_7$ and M$_8$. The black arrow marks the critical time of 3793 fs, where the maximum counterion displacement coincides with the charge disproportionation event.

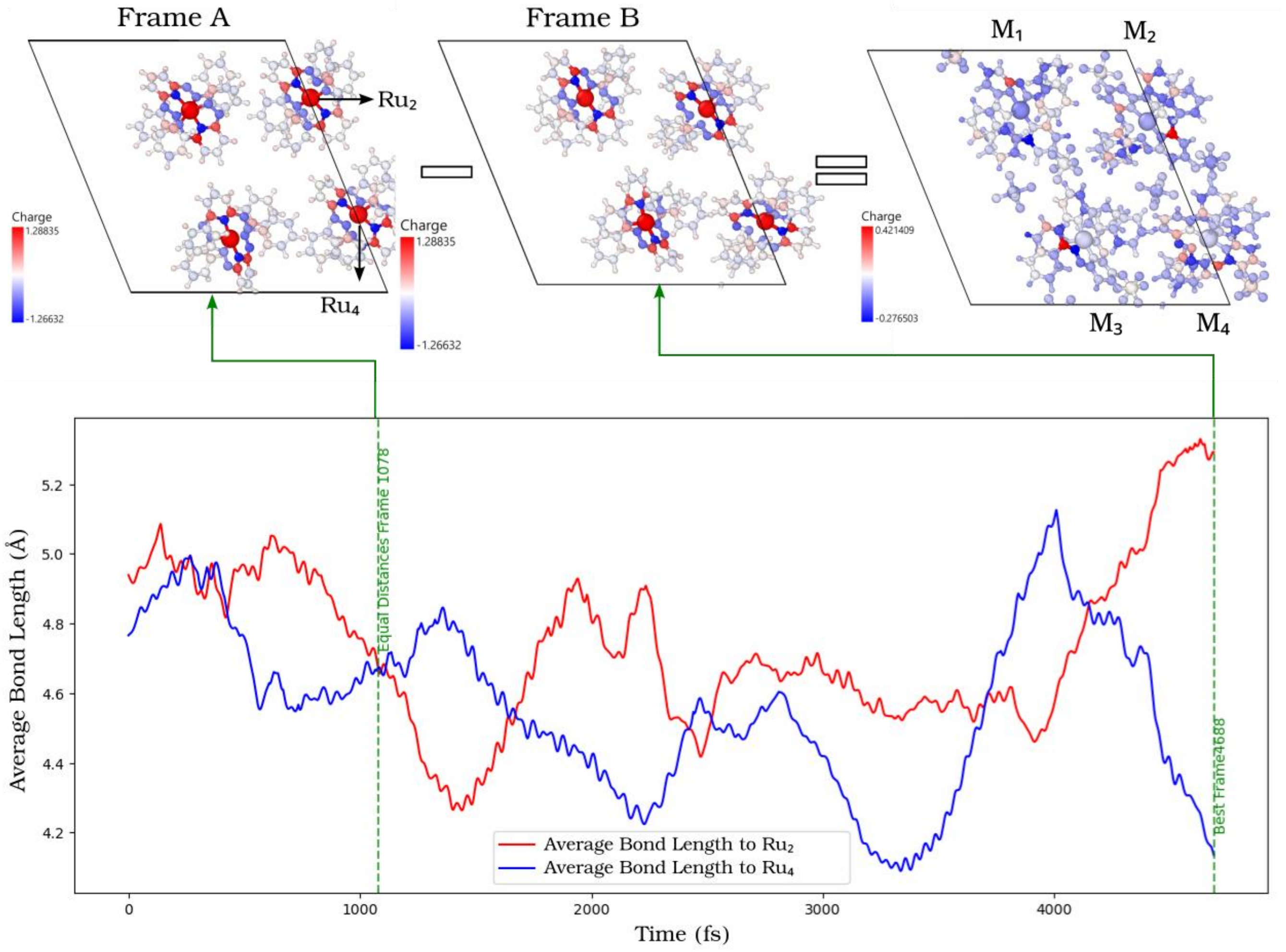

Figure S-9. Dynamic counterion proximity and its impact on electronic charge redistribution in AIMD simulation of the film in State (22) at 750.0 K.

Relative Stability Analysis: State (22) vs. State (13)

The stabilization energy is calculated as the energy of a unit cell containing four molecules after 10,000 fs of molecular dynamics simulation, where the phenomenon of charge delocalization (CD) is identified in all four molecules, minus the energy of the initial system, where the four molecules have a symmetric charge distribution suggesting that each is in the (22) state. This energy difference reflects the driving force for the transition to the asymmetric (13) state, highlighting the role of inter-fragment charge transfer (IFCT) in stabilizing the charge-disproportionated configuration. The stabilization is further supported by constrained DFT (cDFT) calculations performed in the gas phase using NWChem, which reveal a relative energy difference of 1.20 eV, favoring the asymmetric (13) state over the symmetric (22) state.

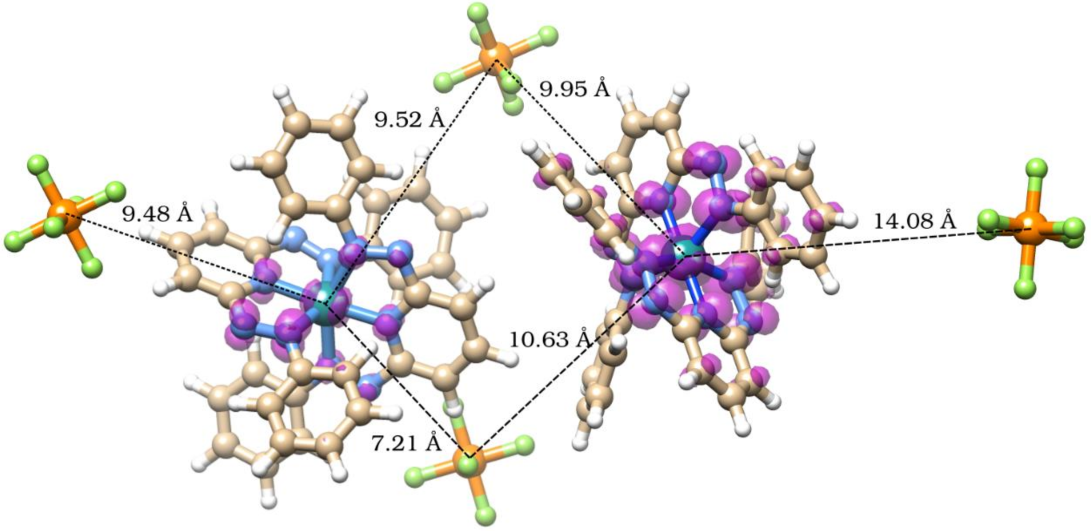

Figure S-10. Optimized gas-phase structure of State (13) obtained via the constrained Density Functional Theory (cDFT) method. An isosurface of α-spin density (isovalue 0.002 e^4/bohr^4) highlights the asymmetric electronic density of unpaired valence electrons. The Ru–P distances illustrate the relative separation between counteranions and the metal centers of the molecules. The fragment on the left, containing one α electron, exhibits shorter Ru–P distances compared to the right fragment, which accommodates three α electrons primarily localized on azo groups (-N=N-) and displays larger metal-to-counteranion distances.

Table S1 - Effect of Counter-Ion Distance on Charge Disproportionation: *M2 and M4 Bader charge differences in frames where counter-ions are equidistant (first four) and in a frame with an unequal counter-ion–metal average bond distance (4688 fs in Fig. S-9). Equidistant counter-ions induce minimal charge difference in the molecules.*

**Time (fs)**	**M2-M4 Charge differences (e^-)**
1249	0.102
1457	0.085
3773	0.067
4107	0.066
4688	0.287

References

(1) Lu, T. A Comprehensive Electron Wavefunction Analysis Toolbox for Chemists, Multiwfn. J. Chem. Phys. 2024, 161 (8), 082503. DOI: 10.1063/J. Chem. Phys.5.0216272.
(2) Lu, T.; Chen, F. Multiwfn: A Multifunctional Wavefunction Analyzer. *J. Comput. Chem.* **2012**, *33* (5), 580-592. DOI: https://doi.org/10.1002/jcc.22885.
(3) Becke, A. D.; Edgecombe, K. E. A Simple Measure of Electron Localization in Atomic and Molecular Systems. *J. Chem. Phys.* **1990**, *92* (9), 5397-5403. DOI: 10.1063/1.458517
(4) Schleyer, P. V.; Maerker, C.; Dranksfeld, A.; Jiao, H.; Hommes, N. J. R. V. Nucleus-Independent Chemical Shifts: A Simple and Efficient Aromaticity Probe. *J. Am. Chem. Soc.* **1996**, *118*, 6317-6318, https://doi.org/10.1021/ja960582d.
